# Assessment of predictive value of thyromental height in predicting difficult laryngoscopy compared with Mallampati, and thyromental distance among surgical patient who will take general anesthesia at selected governmental hospital cross-sectional study: Ethiopia, 2022

**DOI:** 10.1097/MS9.0000000000002388

**Published:** 2024-07-31

**Authors:** Zewetir Ashebir, Fissiha Fentie, Zebiba Mohammed

**Affiliations:** a Department of Anesthesia, School of Medicine, College Of Health Sciences, Addis Ababa University, Addis Ababa; b Wolaita Sodo University, Soddo, Ethiopia

**Keywords:** airway, difficult laryngoscopy, thyromental height test

## Abstract

**Background:**

Anesthesiologists must always perform a preoperative airway examination to identify individuals who will have challenging laryngoscopy. In an effort to identify the most accurate airway predictor, numerous authors have evaluated a number of predictive assays. Thyromental height test (TMHT), a new airway predictor, has recently been demonstrated to have good predictive value in determining difficult airways. This study’s main objective was to assess the diagnostic effectiveness of the TMHT and compare it to other known airway predictors, such as the modified Mallampati test (MMT) and the thyromental distance (TMD).

**Objective:**

To assess the predictive value of TMHT in predicting difficult laryngoscopy compared to modified MMT and TMD among patients who will take general anesthesia.

**Method:**

In this prospective, observational study, which took place from March 2021 to May 2021, 247 people of either sex who were older than 18 but not more than 65 and scheduled for elective surgery under general anesthesia participated. The receiver operating characteristic (ROC) curve was used to identify the proper cut-off values for TMHT, and the Fisher exact test was used to calculate the correlation.

**Result:**

Incidence of Difficult laryngoscopy was 13.4%. For TMHT the cut-off values were 4.9 cm and it had a sensitivity of 78.8%, a specificity of 89.7%, a positive predictive value (PPV) of 54.2%, and a negative predictive value (NPV) of 96.5%, respectively. When compared to other parameters, like TMD, and MMT. TMHT had the highest sensitivity, specificity, PPV, and NPV. (*P*<0.000)

**Conclusion and recommendation:**

Of all the airway assessments, the TMHT had the highest accuracy and odds ratio for predicting difficult laryngoscopy with the highest odds ratio and accuracy. TMHT has to be validated in broader patient groups because it appears to be a possible single anatomical marker for predicting the likelihood of a difficult laryngoscopy. It needs more research because it isn’t assessed in pediatrics and pregnant women.

## Introduction

Highlights

- Incidence of difficult laryngoscopy is 13.4%.
- For thyromental height test (TMHT) the cut-off values is 4.9 cm.
- TMHT had a sensitivity of 78.8%, a specificity of 89.7%, a positive predictive value (PPV) of 54.2%, and a negative predictive value (NPV) of 96.5%, respectively.
- When compared to other parameters, like thyromental distance (TMD), and modified Mallampati test (MMT). TMHT had the highest sensitivity, specificity, PPV, and NPV. (*P*<0.000).

One of the most critical steps in conducting anesthesia is securing the airway. Failure to successfully secure airway may lead to fatal consequences^1^. The most common complication is difficulty in airway. When we say difficult in airway it is defined as to challenging or impossible mask ventilation (IMV), glottis visualization, and/or endotracheal tube placement^2^. Difficult laryngoscopy and intubation are intrinsically linked to poor glottis view; the less suitable the glottis view, the more difficult the intubation^3^. Ease of direct laryngoscopy can be assessed using the Cormack–Lehane system we can grade it from 1 to 4^2,3^, Cormack–Lehane (C–L) view grade 3 (epiglottis only visible) and grade 4 (no glottis structures at all visible) are profoundly connected with troublesome in intubation^3^.

Preoperative evaluation of different anatomic and clinical components makes a difference in distinguishing possibly troublesome^3,4^. Anesthetists can utilize a variety of procedures to determine the complexity of the airway. However, the search for the ideal and generally acceptable classification system continues^5^. In the preoperative phase, widely used tests include the modified Mallampati classification and thyromental distance^6^ that can suggest difficult laryngoscopy and intubation. Mallampati classification was created by Mallampati in the 1960s and then modified by Samsoon in 1987^7^.

The modified Mallampati classification has four classes. MMC is assigned after examining the oropharyngeal structure. The patient is made to sit up straight with their heads in a neutral position to achieve this. The patient next extends his tongue and widens his mouth^7^. If the uvula, tonsillar fauces, hard palate, soft palate, and pillars are all visible, the modified Mallampati class is assigned as Class I; Class II: If the hard palate, soft palate, fauces, and uvula are visible; Class III: If the hard palate, soft palate, and base of the uvula are visible; and Class IV: If the hard palate is the only thing that is visible and the soft palate is not at all visible.. MM Class III and IV are considered as difficult laryngoscopy^6–9^

The thyromental distance (TMD), which must be precisely measured with a ruler, is the distance, when the head is completely extended, between the chin (mentum) and the top of the thyroid cartilage notch. And it’s considered to imply

A TMD measurement of 6.5 cm or more with no other abnormalities suggests that intubation is likely to be easy.
TMD readings between 6.0 and 6.5 cm suggest that laryngoscopy may be difficult as well as alignment of the pharyngeal and laryngeal axes will be difficult.
A TMD measurement smaller than 6 cm suggests difficult laryngoscopy, and intubation may be impossible.

The majority of accessible bedside airway evaluation tests necessitate either patient cooperation (as in the modified Mallampati test, thyromental distance, and sternomental distance) or the presence of teeth (as in the inter-incisor gap and upper lip bite test). In addition, a combination of two or more tests is required^10,11^. Given the limitations of regularly used airway screening examinations, rising evidence suggests the relevance of more recent screening examinations. Interestingly, the thyromental height test was discovered^12^. In search of a single test that is highly accurate in predicting difficult laryngoscopy a current technique was outlined by Etezads *et al.* ^13^. The thyromental height test (TMHT) is a simple and non-invasive procedure. The test is based on the height between the anterior border of the mentum and the thyroid cartilage, while the patient lying flat with the mouth closed and less than 5.1 was indicates difficult in laryngoscopy^12^ currently in different hospitals they use a combination of test to predict difficulty in intubation.

The rate of difficult laryngoscopy is not the same as authors expressed it in several papers. This is often due to the change in patient characteristics like ethnicity^4,14^ so there might be change in the incidence of difficult laryngoscopy and sensitivity, specificity, PPV and NPV so it’s important to know this value to avoid potential harm.

Even though we have different airway assessments, they cannot predict difficult intubation accurately, and there is no single test with high sensitivity and specificity. It would be ideal if we could find a single test with high sensitivity and specificity, so it is critical to have a good predictor of difficult intubation with better sensitivity and specificity.

The TMHT appears promising as a single airway assessment tool to predict the possibility of difficult laryngoscopy^14^. Changes in patient characteristics owing to race or ethnicity may influence the probability of difficult laryngoscopy and difficult intubation^4^. So, it is beneficial to conduct this research at Tikur Anbessa Specialized Hospital and Minilik II hospital to determine the predictive value of TMHT in predicting difficult intubation and to compare it to other tests such as MMT, TMD, and SMD in predicting difficult in laryngoscopy.

As a result, the goal of this study was to see the predictive value of TMHT and how well preoperatively calculated MMC, TMD and TMHT matched with Cormack and Lehane grade during laryngoscopy, and how common difficult laryngoscopy were in individuals who had no obvious difficult airway sign. The rate of difficult laryngoscopy is not the same as authors expressed it in several papers. The prevalence of difficult laryngoscopy differs from what writers have claimed in other papers. This can modify the incidence of difficult laryngoscopy and the sensitivity, specificity, PPV, and NPV, thus it’s crucial to know this number to prevent potential harm. This is frequently caused by changes in patient characteristics like ethnicity^4,14^.

## Methods

An institutional-based cross-sectional study was carried out in Addis Ababa, Ethiopia, between December and April 2022 at selected governmental hospitals. This study has been reported in line with the STROCSS criteria^15^.

### Study setting and period

This study was conducted in Addis Ababa, Ethiopia at Tikur Anbessa Specialized Hospital and Minilik II hospital, which are the largest teaching and referral hospital in the country. In 1972, TASH was founded. It has about 700 beds and annually sees about 250 000 outpatients and 24 000 inpatients, respectively. General surgery, obstetrics and gynecology, neurosurgery, urology, cardiothoracic, pediatric, and orthopedics are among the different departments that are housed there. It has five surgical wards (general surgery), as well as other units for different specialties. Ethiopia’s Menelik II Hospital is a public hospital in Addis Ababa. It is one of the referral hospitals run by Ethiopia’s capital, Addis Ababa. With a catchment area of more than 15 million people, it serves as both a referral and teaching hospital. The research took place between December and April 2022.

### Study design

The study design was an institutionally based prospective cross-sectional study.

### Source population

All patients who underwent elective surgery under general anesthesia at selected governmental hospital were selected.

### Study population

People who underwent general anesthesia with an endotracheal tube and fulfilled the inclusion criteria were included in the study population.

### Dependent variables

Difficult laryngoscopy

### Independent variables

TMHT, MMT, TMD, socio-demographic data, types of surgery, BMI

### Operational definitions

Difficult laryngoscopy: Inability to visualize the vocal cord during direct laryngoscopy after induction and muscle relaxation. CLG III and IV are considered as difficult laryngoscopy.

Prayer’s sign: Also known as diabetic stiff hand syndrome. It is inability of approximating one or more of the digits when the patient attempts to approximate palmar surface of the proximal and distal interphalangeal joints with palms pressed together and digits abducted.

Fixed atlantooccipital joint: Inability of atlantooccipital joint (the joint between the occipital bone and the first cervical vertebra) to slide.

ASA classification: This is patient’s physical status evaluation and based on the physical status of the patient it has six classes: class I, II, III, IV, V, and VI.

BMI: Weight of the patient divided by height square. This is important to classify the patient as obese, overweight or normal

MMT: This test has four grades, so the score can be between 1 and 4 points. Mallampati scores of 3 and 4 are considered predictive of a difficult intubation. Patients are asked to make an “a” sound without phonation while opening the mouth, and the pharyngeal structures are visualized with the head in slight extension.

TMD: The head is fully extended, and the distance between lower border of the mandibular mentum and thyroid protrusion is measured along a straight line. The short thyromental distance (TMD ≤6.5 cm) has been correlated with difficult direct laryngoscopic intubations in adult patients.

Endotracheal tube: A tube use to insert into the trachea to provide artificial ventilation.

General anesthesia: It is medicine that is administered by professional anesthetist or anesthesiologist through a mask or an IV placed in the vein. While the anesthesia is working the patient will be unconscious, and most of the body functions will slow down or need help to work effectively.

TMHT: the height between the anterior border of the thyroid cartilage (on the thyroid notch just between the 2 thyroid laminae) and the anterior border of the mentum (on the mental protuberance of the mandible)

Sensitivity (SE): The conditional probability of correctly identifying difficult laryngoscopy by TMHT, MMT, AND TMD se=TP/TP+FN

Specificity (SP): The probability of correctly diagnose or identify not being difficult laryngoscopy by TMHT, MMT, TMD. SP=TN/TN+FP

Positive predictive value (PPV): The probability of being difficult airway for TMD, TMHT, and MMT predicted difficult laryngoscopy. PPV=TP/TP+FP

Negative predictive value (NPV): It is the probability of not being difficult airway for MMT, TMD, TMHT predicted not being difficult laryngoscopy. NPV= TN/TN+FN

### Inclusion criteria

Age of 18–65 years, ASA class I and II, Patients taking general anesthesia with ETT and BMI less than 30

### Exclusion criteria

Patients with apparent restriction of mouth opening, DM patients with prayers sign, patients with immobile atlantooccipital joint and cervical vertebrae, patient with oral mass, patient with maxillofacial trauma, BMI greater than 30, large anterior neck mass like huge goiter, airway malformation, burn contracture, patient who need awake fiberoptic intubation, Edentulous patient

Sample size determination

Sample size was determined using the finite population correction formula by assuming the prevalence as 0.5 and 5% margin of error at the 95% CI using the following formula


$$n=\frac{\left(Z\alpha /2\;)2P(1-p\right)}{w2}$$

Where; *n* = sample size, z= 1.96, *P*= 0.5, w= 0.05, CI= 95% & ἀ= 5%


$$n=\frac{\left(1.96\right)2\;\times \;0.5\;\left(1-\;0.5\right)}{0.05\times 2\;=\;384}$$

*nf* = *n*/ (1+*n/N*), *N*=531 ( *N*_TASH_=97, and *N*_MH_=80) per month from situational analysis and log book review)

Where n denotes the total sample size and *N* denotes the population per month.

So, *nf* =384/ (1+384/531)=223 10% of for non-response rate will be added; (i.e. 223+24=247

Therefore, a total sample size of 247 elective surgical patients will participated in this study.

## Sampling technique

**Figure 1:**
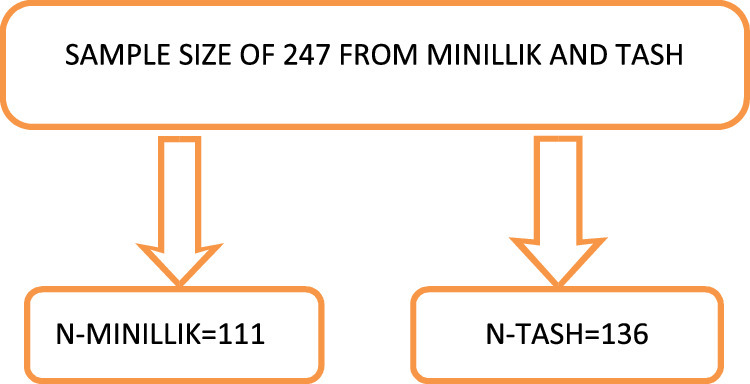
Proportion of sample size between hospitals.

A systematic random sampling procedure was used to collect the necessary number of samples during the course of the study period. We used the daily operation schedule list as a sampling window. Ninety-seven elective procedures at TASH and 80 at Minilik II Hospital were performed over the course of three months, according to scenario analysis and log book inspection. When the samples are distributed according to proportion, 111 patients will be drawn from Minilik II hospital and 136 from TASH hospital, as shown in Figure 1. Using the equation *K*=*N/n*, where 247/177 is 1.4, *K* was calculated. Where *N* is the monthly population and *n* is the sample size overall. The necessary sample size was attained during the study period by using a systematic random sampling procedure. We used the daily operation schedule list as a sampling frame. As a result, the sampling interval was set at two, and the first study participant was chosen by lottery from the list of the daily operating schedule participants.

### Data collection technique

Data collectors and supervisors received training on all of the study instruments’ components as well as the full data collection process, study goals, and relevance for one day. The data collection method (the questionnaire) was pre-tested on 13 patients who were not part of the main experiment, and regular supervision and follow-up were conducted during data collection. Every day, a supervisor verified each questionnaire for completeness and consistency of data, which was then double-checked by the primary investigator. There was no data that was missing.

Then, after obtaining signed informed consent, preoperatively, two trained anesthetists inspected the airway TMHT was assessed using depth gauge when the patient was supine, for TMD we used rigid ruler and MMC was assessed by visualization when the patient was at sitting position. Before surgery, all of the patients were fasted for 8 hours. Anesthetist who was not involved in airway assessment did a laryngoscopy in the sniffing position with a Macintosh #3, 4 blades. The patient’s trachea was subsequently intubated, with bilateral auscultation over the lung fields. The modified Cormack and Lehane (CL) grading system was used to evaluate the laryngeal view.

## Data quality control

### Data collectors were trained by principal investigator

For data collecting, trained data collectors and a supervisor were assigned. Data were collected in selected study participants from February to April 2021 using a pre-tested questionnaire. All materials used for data collection were organized sequentially, and data was stored in a safe and secure location.

### Data analysis and interpretation

Data was checked, coded, entered and analyzed by using SPSS version 26. Demographic data was Patient data were presented as mean±standard deviation or numbers (%). Area under the receiver operating characteristic (ROC) curve and area under the curve (AUC) were used to calculate the optimal predictive cut-off point for TMHT, the preoperative airway assessment data and the findings during intubation were used to calculate the validity indexes. Fischer exact test was used for statistical comparison; 95% CI was calculated; and a *P* value of 0.05 (two-tailed) was defined as statistically significant. Sensitivity and specificity, positive and negative predictive value of TMH, MMT in relation to CLG was calculated by cross tabulation.

## Results

In this study, 247 patients were included. Demographic data such as age, weight, BMI, and height were expressed using mean, MIN, MAX, and SD. Table 1 shows that patients from various specialties were included, with urology surgery having the most patients, followed by gynecology, endocrine, ENT, and hepatobiliary having the fewest. There were also more males than females, with 129 and 118, respectively.

**Table 1:** Demographic and clinical characteristics of the study population.

Variable	Age (year)	Weight (kg)	BMI (kg/m2)	Height (cm)	Sex
Mean	39.4	62.6	23.4	1.64	Male (52.2%)
Min	18	45	16.7	1.5	Female (47.8)
Max	66	83	30.5	1.8	
SD	10.9	7.2	2.6	7.2	

Max, maximum; Min, minimum.

**Figure 2:**
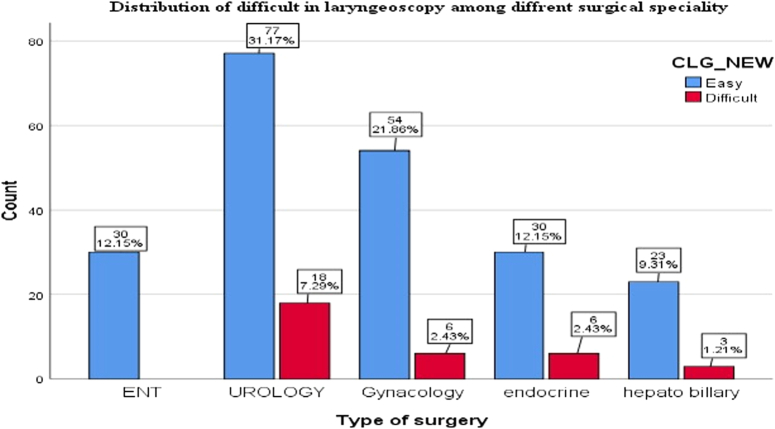
Distribution of different surgical specialties with occurrence of difficulty in laryngoscopy.

The incidence of difficult laryngoscopy was found to be 13.4% from all patients, with the highest number recorded in urology surgery, followed by gynecology and endocrine surgery, and then hepatobiliary surgery, with an incidence of 18%, 6%, 6%, and 3%, respectively, while there was no data recorded in ENT surgery, as shown in Figure 2.

**Figure 3:**
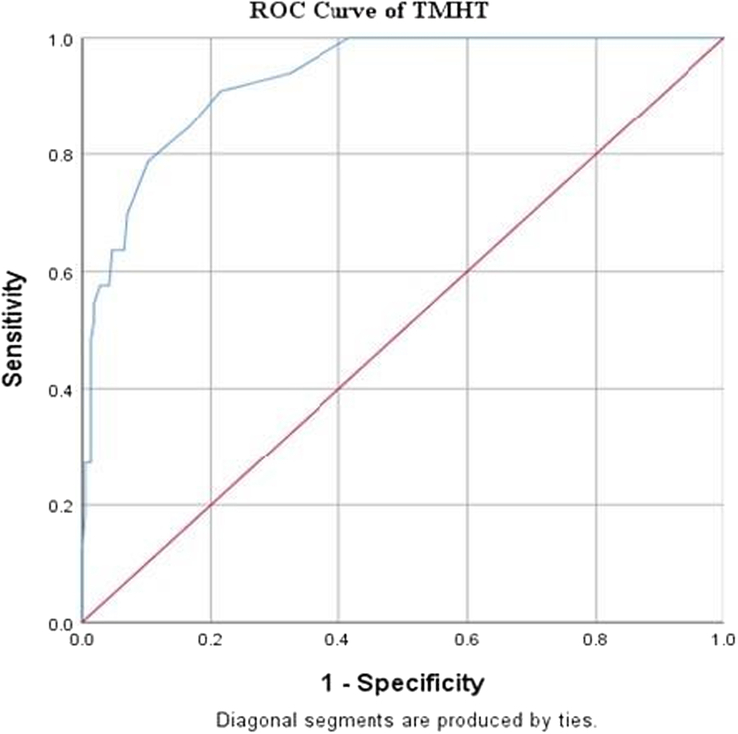
ROC curve for TMHT. ROC, receiver operating characteristic; TMHT, thyromental height test.

Three airway parameters were recorded prior to surgery: TMHT, MG, and TMD. The sensitivity and specificity of these parameters were calculated, and for TMHT, the cut-off value was determined to be 4.9 using the ROC curve, with an AUC of 0.93, sensitivity of 78.8, and specificity of 89.9. The three airway parameters’ accuracy, OR, RR, PPV, and NPV were also calculated, and the results are shown in Table 2 and Figure 3.

**Table 2:** Comparison between CLG grades and three preoperative predictors (TMHT, MMT, and TMD).

Test	CLG-I and II	CLG III and IV	Total	Sign (Fisher exact test)
TMHT				
<4.9	22	26	46	0.000
>4.9	192	7	199	0.000
MMT				
I and II	204	19	223	0.000
III and IV	10	14	24	0.000
TMD				
<6.5	39	13	52	0.010
>6.5	175	20	195	0.010

CLG, Cormack and Lehane Grading; MMT, modified Mallampati test; TMD, thyromental distance; TMHT, thyromental height test.

**Figure 4:**
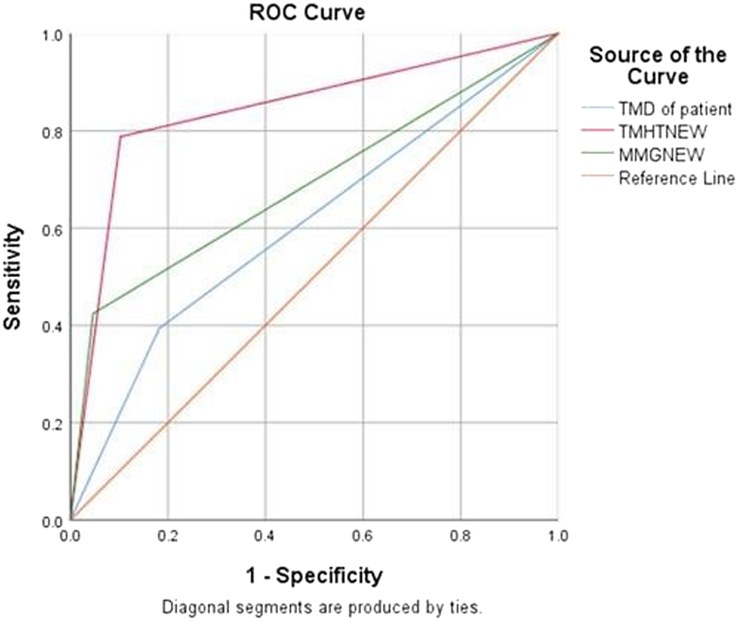
ROC curve for TMHT, MMC, and TMD. MMC, modified Mallampati classification; ROC, receiver operating characteristic; TMD, thyromental distance; TMHT, thyromental height test.

With a cut-off value of 4.9, 199 of the patients had CLG of greater than 4.9, with 7 of them having CLG of I and II and the remaining 48 patients having TMHT of 4.9 and 26 having difficulty with laryngoscopy, as shown in Table 3 and Figure 4.

**Table 3:** Validity indexes for TMHT, MMT and TMD to predict the occurrence of difficult laryngoscopy

Parameters	Sn (%)	Sp (%)	PPV (%)	NPV (%)	AUC	RR	*P* value (Fisher exact test)	Accuracy (%)	OR
TMHT	78.8	89.7	54.2	96.5	0.930	13.53	0.000	88.3	32.9
MMT	42.2	95.3	58.3	91.5	0.689	7.04	0.000	88.3	14
TMD	39.4	81.8	25	89.7	0.606	2.93	0.010	76.1	2.63

AUC, area under the curve; MMT, modified Mallampati test; NPV, negative predictive value; OR, odds ratio; PPV, positive predictive value; RR, relative risk; TMD, thyromental distance; TMHT, thyromental height test.

## Discussion

Anesthesiologists must always perform a preoperative airway examination to identify individuals who will have challenging laryngoscopy. It has recently been shown that the TMHT, a novel airway predictor, has good predictive value for identifying challenging airways. Based on our research, TMHT has the highest accuracy and odds ratio for predicting difficult laryngoscopy. Our findings indicate that the TMHT cut-off value is 4.9, and the incidence of difficult laryngoscopy is 13.4%.

The results of our study showed that the sensitivity, specificity, PPV, and NPV of TMHT (78.8%, 89.7%, 54.2%, and 96.5%), MMT (42.4%, 95.3%, 58.5%, and 91.5%), and TMD (39.4%, 81.8%, 25%, and 89.7%) are higher than those of MMT and TMD. MMT was better than TMD in terms of specificity, PPV, and NPV but had a lower sensitivity. While specificity and NPV were high (42.4%, 95.3%, 58.5%, and 91.5%, respectively), TMD had the lowest sensitivity and PPV.

The incidence of difficult laryngoscopy, which we found to be 13.4% in our study, is close to other literature, which reports similar incidences ranging from 1.3 to 13%^6,15–18^. The TMHT cut-off value of 4.9 was discovered; this is rather less than the initial research, which was suggested by Etezadi *et al.* ^14^ (5 cm). This could be because of racial and population differences in the population.

With a cut-off value of less than 5 cm, Etezadi *et al.* ^14^ calculated sensitivity and specificity of 83.6% and 99.3 respectively, PPV of 90.47% and NPV of 98.3%, and we found sensitivity and specificity of TMHT to be 78.8 and 89.7, respectively, with PPV of 54.2% and NPV of 96.5. These results are somewhat lower than ours. The sensitivity, specificity, and NPV (84.8%, 83.2%, and 97.3%) will all increase if we raise our cut-off value, but the PPV (43.8%) will decrease since we are more interested in differentiating between tough and easy laryngoscopies.

In the Turkish study, the cut-off value for TMHT was found to be less than 4.3 cm, which is lower than in both our study and the original study. Our study yielded comparable results in terms of specificity and NPV, with a value of 96.13 and 78.02%, but lower sensitivity and PPV, at 64.86 and 20.87, respectively^19^. A cut-off of 52.17 was also determined in India, yielding a sensitivity of 81.25% and specificity of 92.33%^20^.

According to Pratibha *et al.* ^21^, he discovered that the TMHT’s sensitivity, specificity, PPV, and NPV were, respectively, 78.18%, 93.94%, 58.90%, and 97.48%, with a cut-off value of 5 cm. The original literature by Etezadi *et al.* suggested this cut-off value. Despite the fact that their reduction of value was marginally smaller than our report, our outcome remained the same.

Using the Fisher exact test, the association between TMHT and difficult laryngoscopy was evaluated. The results showed a substantial correlation with a *P* value of less than 0.000, which is comparable to Pratibha’s and others^21^

Despite the fact that different literature used different cut-off values for their TMHT and discovered inter-observational contrasting values for sensitivity, specificity, PPV and NPV, they all agreed that TMHT provided better sensitivity, specificity, PPV and NPV than other airway parameters^1,11,12,20^, which is equivalent to our finding.

In contrast to our findings and the initial Etezadi and colleagues result, Siddanagouda reported poor sensitivity, specificity, and accuracy for TMHT of 50%, 57.14%, and 56.6%, respectively. They conclude in their study that TMD and MMC appear to be better predictors of difficulty in laryngoscopy than TMHT^13^. This discrepancy may be due to the fact that all patients had assessments while supine, which inherently results in cervical extension; the patient’s compliance dictated the extent of cervical extension when the mouth was closed. As the patient stretched his head more, TMH increased. This led to a false negative test, which is extremely harmful for airway management^13^. Additionally, minimal neck flexion during evaluation may increase the number of false positive results.

In a study carried out by Tamirat, it was also discovered that the sensitivity specificity NPV and PPV of MMC to were 42.4%, 95.3%, 91.5, and 58.5, respectively, with an AUC of o.689.They discovered that, in line with our data, the sensitivity was 45.8%, while the specificity, PPV, and NPV were lower at 65.9%, 20.4%, and 86.2%, respectively^22^.

Tadese also reported a greater sensitivity of 51.5% along with specificity and PPV of 94.7% and 60.7%, respectively. Additionally, according to studies by Sagar and colleagues, the sensitivity, specificity, and NPV of MMT are 50%, 89.28%, and 96.15%, respectively. This differs from our findings, which showed higher sensitivity and PPV but lower NPV from our study^13^. Our population differences could be the cause of this variation.

The research conducted by Pratibha did not differ from our findings^21^, and other literature also suggests that TMD is a list predictor of difficulty in laryngoscopy when compared with other airway parameters. In our study, TMD is the list predictor of difficulty in laryngoscopy than MMC and thyromental height test with low sensitivity, PPV OF 39.4%, 25% and specificity, NPV of 81.8%, 89.7%, respectively^6,10,22^.

According to Alemayehu *et al.* ^22^, TMD has extremely low sensitivity specificity, PPV, and NPV, which are 58%, 22%, 12.6%, and 73%, respectively. As a result, TMD is one of the tests that predict laryngoscopy difficulty and has a higher false positive rate than other tests. Because it is on the list of predictive tests with low sp, PPV, and NPV, it is comparable to our study and Tamire *et al*.^6^ study.

### Limitation of study

- Due to experience, there may be inter-observational differences in laryngoscopic view because the research was conducted in a teaching institution.

### Strength of study

- Participants were homogenous.
- The strength of this study is that all patients who fulfilled the inclusion criteria in the study period were included in the study.
- As far as we are aware, no study has been conducted to evaluate the predictive value of TMHT and compare it with other airway parameters that are difficult to predict during laryngoscopy; additionally, this is a multi-center investigation. It therefore serves as a springboard for additional research.

## Conclusion and recommendations

### Conclusion

Our research shows that TMHT is the best predictive test for difficult laryngoscopy, with the highest sensitivity, PPV, NPV, and odds ratio of any predictive test studied. TMHT is also a simple bedside test that does not require the patient to extend his or her head. TMHT has lower interobserver variability and a higher degree of accuracy when compared to other predictive tests.

### Recommendation

TMHT appears to be a promising single anatomical measure for predicting the likelihood of difficult laryngoscopy, but it must be verified in larger patient populations.

It will require additional examination because it has not been investigated in pediatrics or pregnant mothers.

## Ethical approval

Ethical clearance was obtained from the ethical clearance committee of Addis Ababa University, department of anesthesia and permission was obtained from Tikur Anbessa Specialized Hospital and Minilik II hospital before the start of the study.

## Consent

Not applicable.

## Source of funding

Addis Ababa University.

## Author contribution

Z.A., F.F. and Z.M. and: as a team developed the proposal, trained the data collectors, analyzed the data and prepared the manuscript.

## Conflicts of interest disclosure

The authors declare no conflict of interest.

## Research registration unique identifying number (UIN)

This study is registered at http://www.researchregistry.com with Registration number: with Registration number: researchregistry10056.

## Guarantor

Zebiba Mohammed, Zewetir Ashebir, Fissiha Fentie.

## Data availability statement

Data and materials will be shared upon reasonable request.

## Provenance and peer review

The paper was reviewed and presented at the department level.
